# Supplementary material for: Identification of photoperiod-induced specific miRNAs in the adrenal glands of Sunite sheep (Ovis aries)
Source: Front Vet Sci. 2022 Jul 22;9:888207. doi: 10.3389/fvets.2022.888207 (PMC9354845; doi:10.3389/fvets.2022.888207)
Supplement: Supplementary file 1 [file Data_Sheet_1.ZIP › Supplementary file/miRDeep2 file.zip!/miRDeep2 file/Know_Structure/SPLP42A2/oar-mir-376c.pdf]

miRBase precursor : oar-mir-376c
Total read count : 486
oar-miR-376c-5p read count : 189
oar-miR-376c-3p read count : 296
remaining reads : 1

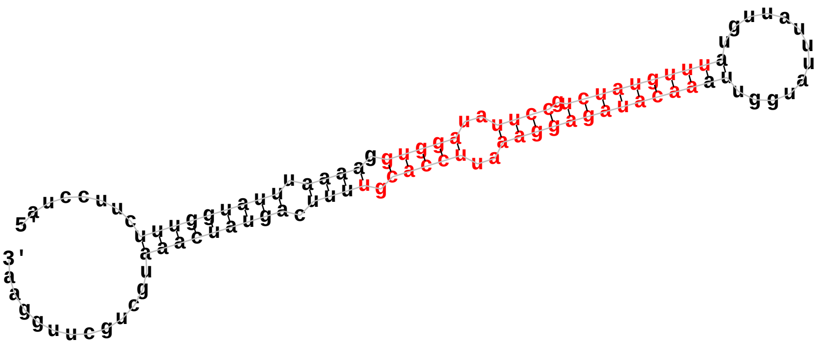

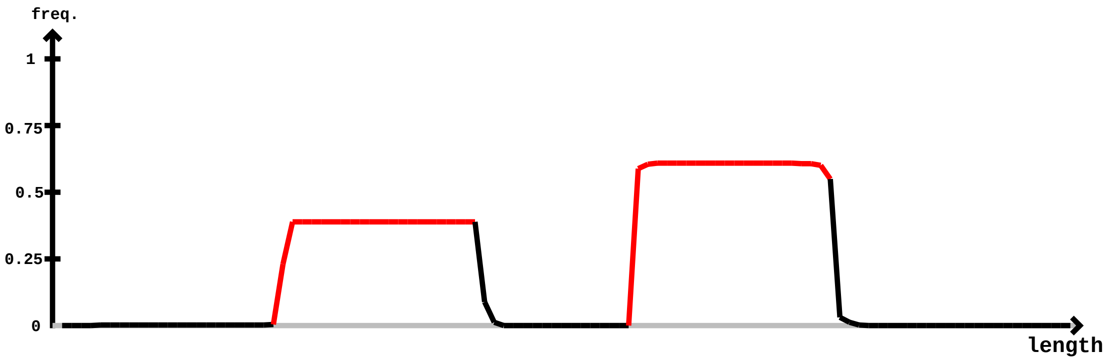

oar-miR-376c-3p
oar-miR-376c-5p

```
5'- auccuucuuugguauuuaaaagguggauauuccgucuauguuuauguuauuuaugguuaaacauagaggaaauuccacguuuucaguaucaaaugcugcuuggaa  -3'   exp
    .......((((((((.((((.(((((..((((.(((((((((((............))))))))))))))..))))).)))).))))))))).............   reads   mm   sample
    ...cuucuuugguauuuaaaag...................................................................................   1       0    seq
    .....................gguggauauuccgucuauguuu..............................................................   1       0    seq
    ......................guggauauuccgucuauguuu..............................................................   87      0    seq
    ......................guggauauuccgucuauguuua.............................................................   23      0    seq
    ......................guggauauuccgucuauguuuau............................................................   1       0    seq
    .......................uggauauuccgucuauguuu..............................................................   58      0    seq
    .......................uggauauuccgucuauguuua.............................................................   14      0    seq
    .......................uggauauuccgucuauguuuau............................................................   5       0    seq
    ...........................................................aacauaAaggaaauucc.............................   1       1    seq
    ...........................................................aacauagaggaaauuccac...........................   3       0    seq
    ...........................................................aacauagaggaaauuccacg..........................   25      0    seq
    ...........................................................aacauagaggaaauuccacgu.........................   249     0    seq
    ...........................................................aacauagaggaaauuccacguu........................   7       0    seq
    ...........................................................aacauagaggaaauuccacguuu.......................   1       0    seq
    ............................................................acauagaggaaauuccacgu.........................   3       0    seq
    ............................................................acauagaggaaauuccacguu........................   2       0    seq
    ............................................................acauagaggaaauuccacguuu.......................   3       0    seq
    .............................................................cauagaggaaauuccacguuu.......................   1       0    seq
    .............................................................cauagaggaaauuccacguuuu......................   1       0    seq
```
